# Accurate Measurement of Mitochondrial DNA Deletion Level and Copy Number Differences in Human Skeletal Muscle

**DOI:** 10.1371/journal.pone.0114462

**Published:** 2014-12-04

**Authors:** John P. Grady, Julie L. Murphy, Emma L. Blakely, Ronald G. Haller, Robert W. Taylor, Doug M. Turnbull, Helen A. L. Tuppen

**Affiliations:** 1 Wellcome Trust Centre for Mitochondrial Research, Institute of Neuroscience, Newcastle University, Newcastle upon Tyne, United Kingdom; 2 Department of Neurology, University of Texas Southwestern Medical Center and VA North Texas Medical Center, and Neuromuscular Center, Institute for Exercise and Environmental Medicine, Dallas, Texas, United States of America; Leibniz Institute for Age Research - Fritz Lipmann Institute (FLI), Germany

## Abstract

Accurate and reliable quantification of the abundance of mitochondrial DNA (mtDNA) molecules, both wild-type and those harbouring pathogenic mutations, is important not only for understanding the progression of mtDNA disease but also for evaluating novel therapeutic approaches. A clear understanding of the sensitivity of mtDNA measurement assays under different experimental conditions is therefore critical, however it is routinely lacking for most published mtDNA quantification assays. Here, we comprehensively assess the variability of two quantitative Taqman real-time PCR assays, a widely-applied *MT-ND1*/*MT-ND4* multiplex mtDNA deletion assay and a recently developed *MT-ND1*/*B2M* singleplex mtDNA copy number assay, across a range of DNA concentrations and mtDNA deletion/copy number levels. Uniquely, we provide a specific guide detailing necessary numbers of sample and real-time PCR plate replicates for accurately and consistently determining a given difference in mtDNA deletion levels and copy number in homogenate skeletal muscle DNA.

## Introduction

The mitochondrial genome (mtDNA) is a multicopy 16.5 kb circular, double-stranded DNA molecule that encodes 13 essential subunits of the mitochondrial respiratory chain, as well as 22 mt-tRNAs and two mt-rRNAs necessary for their synthesis. A wide variety of phenotypically heterogeneous diseases, frequently involving the neuromuscular system [1], are associated with mtDNA mutations and/or changes in the cellular abundance of mtDNA molecules. MtDNA mutations can vary from point mutations through to extensive genomic rearrangements, with large-scale mtDNA deletions occurring in approximately 13% of patients with mtDNA disease [2]. Both mutated and wild-type forms of mtDNA can coexist within a cell, a situation termed heteroplasmy. When mutation loads exceed a critical threshold level within an individual cell (typically >60%), a biochemical defect, most often a deficiency in the activity of cytochrome *c* oxidase (COX), can be detected [3]. The overall number of mtDNA molecules present can also influence a cell’s respiratory capacity. A quantitative loss of mtDNA copy number of more than 30% of wild-type level has been linked to multisystemic mitochondrial disease [4]. Conversely, an increase in mtDNA abundance can represent a compensatory response to inefficient mitochondrial respiratory function [5].

A key requirement in mtDNA disease research is thus the quantification of mtDNA molecules, both wild-type molecules and those harbouring pathogenic mutations. These measurements must be accurate and reliable, particularly to enable the detection of small differences in mtDNA abundance and integrity in homogenate tissues. This is of crucial importance for elucidating the molecular mechanisms involved in mtDNA pathologies and their clinical progression. Moreover, it is vital for determining the value of novel therapeutic approaches, such as endurance and resistance exercise training regimes [6]–[9].

At present, the most commonly employed technology for the quantitative analysis of mtDNA molecules is real-time PCR. A variety of assays have been published (e.g. singleplex, multiplex, three primer assay), differing in the nuclear and mitochondrial genomic locations they target and the real-time chemistries they exploit [10]–[17]. To the best of our knowledge a comprehensive assessment of the sensitivity of each of these assays, including our own previously described *MT*-*ND1*/*MT*-*ND4* and *MT*-*ND1*/*B2M* assays [11], [18], [19], has never been published. This characterisation is particularly important in light of a recent report on the effect of DNA concentration on measurement error in real-time PCR assays, which describes how empirical error increases as DNA concentration decreases [20]. Moreover, the relationship between sample heteroplasmy and real-time assay sensitivity has never been investigated, despite indications that measurement error increases as deleted mtDNA levels decrease [11].

Here, we detail the variability present in our Taqman *MT*-*ND1*/*MT*-*ND4* real-time PCR assay with respect to two variables: the DNA concentration of homogenate skeletal muscle samples and the level of deleted mtDNA. We also characterise the sensitivity and reproducibility of a Taqman mtDNA copy number assay, which targets *MT*-*ND1* and the nuclear *B2M* gene in separate reactions, under different DNA concentration and copy number conditions. Finally, guidelines for achieving the most accurate and reliable mtDNA measurements at these different levels of mtDNA deletion/copy number and DNA concentration in homogenate skeletal muscle are provided.

## Materials and Methods

This study had relevant ethical approval from the institutional review board of Newcastle University and complied with the Declaration of Helsinki. Written informed consent was obtained from all patients involved.

### DNA samples and handling

DNA samples originated from patients harbouring different loads of single, large-scale mtDNA deletions (6–84% mutant load; *n* = 6) and from control individuals (*n* = 3) with varying mtDNA copy numbers (Table 1). Total DNA had been extracted from skeletal muscle (quadriceps) needle biopsies (approximately 10 mg tissue) using the EZ1 DNA Tissue extraction kit (Qiagen, Crawley, UK), as per the manufacturer’s recommendations. DNA concentrations were determined using a Nanodrop ND-1000 UV-Vis spectrophotometer (Thermo Fisher Scientific). DNA samples were stored at 4°C for the duration of the study (up to 1 month) to avoid freeze-thaw artefacts. The preservation of DNA integrity over this time period was confirmed by agarose gel electrophoresis (Figure S1). All DNA dilutions were systematically performed in 10-fold serial dilution steps, with pipette tips being flushed out 20 times prior to pulse-vortexing the DNA on a medium setting for 15 seconds between each dilution.

**Table 1:** Clinical details of patients with single, large-scale mtDNA deletions and control individuals.

Subject	Sex	Age^1^	Clinicalfeatures	MtDNA deletionbreakpoints^2^	MtDNAdeletionlevel
Patient 1^3^	F	40	CPEO	9486–13723	84%
Patient 2	M	41	CPEO,epilepsy	8469–13447	69%
Patient 3^4^	M	40	CPEO	8289–13041	48%
Patient 4	M	37	CPEO,diplopia,myopathy,fatigue	7637–15676	38%
Patient 5	F	33	CPEO,myopathy,fatigue	9754–15567	20%
Patient 6	M	38	CPEO	7845–15440	6%
Control 1	M	27	-	-	-
Control 2	M	45	-	-	-
Control 3	M	58	-	-	-

CPEO, chronic progressive external ophthalmoplegia.

1 Age at time of biopsy.

2 GenBank accession number NC_012920.1.

3 Patient 1 published in [7].

4 Patient 3 published in [8].

### Mitochondrial DNA deletion assay

The proportion of deleted mtDNA was determined using the previously described multiplex *MT-ND1*/*MT-ND4* Taqman real-time PCR assay [11] on a StepOne Plus real time machine (Applied Biosystems, Warrington, UK), with the following modifications. Reactions were performed in 20 µl volumes. Probes were designed with a non-fluorescent quencher and MGB moiety: *MT-ND1* probe VIC-5′CCATCACCCTCTACATCACCGCCC-3′-MGB (revised Cambridge Reference Sequence (rCRS) location m.3506-3529; GenBank accession number NC_012920.1) and *MT-ND4* probe FAM-5′-CCGACATCATTACCGGGTTTTCCTCTTG-3′-MGB, rCRS location m.12111-12138). Standard curves were included for analysis of data. To minimise pipetting error, DNA from single deletion mtDNA patients was added to each well in 5 µl volumes. Intra-plate variance was investigated using the single mtDNA deletion patient DNA samples at a minimum of three different concentrations (with final PCR DNA concentrations ([DNA]_PCR_) ranging between 0.1 ng/µl and 0.1 fg/µl), with each concentration assayed in 84 replicates on a single real-time plate. To determine the inter-plate variance of the estimate of deletion level differences between samples at a similar deletion level, the 10% and 80% deletion level DNA samples (patients 1 and 6, respectively) were mixed in varying ratios to generate low deletion level (20–25%; *n* = 3, numbered A–C) and high deletion level (70–80%; *n* = 3, numbered D–E) samples, which were analysed in 24 replicates ([DNA]_PCR_>1 pg/µl) on separate real-time plates (*n* = 3). The proportion of deleted mtDNA was calculated from the *MT-ND4* (wild-type molecules) and *MT-ND1* (total mtDNA) data.

### Mitochondrial DNA copy number

Relative levels of mtDNA copy number were determined by real-time PCR using singleplex Taqman assays designed to target the mitochondrial *MT-ND1* gene (as described in mtDNA deletion assay method) and the nuclear *B2M* gene (GenBank accession number: NG_012920). Primers and probe for the *B2M* assay were as follows: *B2M* forward primer 5′-CCAGCAGAGAATGGAAAGTCAA-3′ (gene location n.8969-8990), *B2M* reverse primer 5′-TCTCTCTCCATTCTTCAGTAAGTCAACT-3′ (n.9064-9037) and *B2M* probe 6FAM-5′-ATGTGTCTGGGTTTCATCCATCCGACA-3′-MGB (n.9006-9032). Final *B2M* primer and probe concentrations were 300 nM and 100 nM, respectively. Each 20 µl *B2M* reaction was supplemented with 3 mM MgCl_2_. To minimise pipetting error, DNA was added to each well in 5 µl volumes. Standard curves were included for analysis of data. *MT-ND1* and *B2M* Taqman assays were analysed sequentially on the same real time machine; samples were located in the same wells on paired plates to minimise well-to-well error. To determine intra- and inter-plate variance, two control skeletal muscle DNA samples with high (approximately two-fold) relative copy number difference and of equivalent concentration were mixed in varying ratios to generate samples of progressively increasing mtDNA copy number (numbered S1 to S5). These five samples were analysed in duodecuplicate on each *B2M* and *MT*-*ND1* real-time PCR plate at varying DNA concentrations; each plate was performed in quadruplicate. To ensure the sample quantification cycles (C_q_) for both reactions were within an optimal detection range of 17 to 33, final PCR DNA concentrations differed by an order of magnitude between the mitochondrial (20 pg/µl>[DNA]_PCR_>0.3 pg/µl) and nuclear (0.6 ng/µl>[DNA]_PCR_>10 pg/µl) reactions. The *MT*-*ND1*/*B2M* copy number ratio was calculated for each sample well on each plate, yielding 12 replicate values per sample per *MT*-*ND1*/*B2M* plate pair. The relative copy numbers of samples S2 to S5 compared to sample S1 were calculated from these data, resulting in 48 independent sample comparisons per *MT*-*ND1*/*B2M* plate pair.

### Statistical analysis and data reporting

For each analysis, severe outliers, defined as data points more than three times the interquartile range below or above the lower and upper data quartiles, respectively, were removed from the data prior to further analysis. This approach was adopted in order to remove any operator-induced error in the data. It resulted in the removal of 31 values from the 2352 total replicates (1.3%) generated for determining the intra-plate deletion level variation, 6 values from the 432 replicates (1.4%) obtained for assessing the inter-plate mtDNA deletion level variation and 17 values from the 720 replicates (2.4%) used for calculating the mtDNA copy number assay variation.

Intra-plate variance, for both the mtDNA deletion level and copy number assays, was quantified by the standard deviation of the replicates on each plate and bias-corrected [21]. The large number of replicates generated with the deletion assay permitted the use of bootstrapping to calculate 95% confidence intervals for the standard deviation [22]. Inter-plate variance was assessed by taking the estimate of each measure (either deletion level or relative copy number difference) from each plate and calculating the standard deviation of these values, which were also bias-corrected. The standard deviation estimates were further adjusted to correct for the intra-plate variance by subtracting the estimated intra-plate variance divided by the number of replicates per plate. Bootstrapping was performed in MATLAB Release 2012A (The MathWorks Inc., Natick, MA). SAS version 9.2 (Cary, NC) was used for all other statistical analyses. PROC GLM was used to perform linear regression to investigate the relationship between assay variation, DNA concentration (C_q_), and mtDNA deletion level/copy number.

Where linear regression is used the correlation coefficient (r), number of independent samples (*n*) and P-value are reported. Parameter estimates are reported separately where appropriate. Where the relationship between variables is non-linear, the Spearman’s rho (Spearman’s r), *n* and P-value are reported.

The number of repeat plates (with 3 or 6 replicates per sample per plate) required to observe a given deletion level or relative copy number difference was calculated from the estimated inter- and intra-plate standard deviations using a two tailed t-test at β = 0.80 and α = 0.05. For the *MT-ND1*/*MT-ND4* assay, only data obtained from the high DNA concentration reactions were used, exploiting the observed linear relationship between standard deviation and target deletion level. For the mtDNA copy number assay, the standard deviations at low, moderate and high PCR DNA concentrations were used to estimate the number of plates required. The standard deviations are reported in percentage points for the deletion level assay. For the copy number assay, the error is linearly related to the log of the mtDNA copy number values, and thus the geometric standard deviation is reported; equivalent scale factors (expressing percentage change) were calculated by taking the exponential of the geometric standard deviation.

## Results

Quantification of variability within the *MT*-*ND1*/*MT*-*ND4* and *MT*-*ND1*/*B2M* real time PCR assays requires measurement of both the precision and the accuracy of the assays. Intra-plate variability is a straightforward estimate of the precision. Similarly, inter-plate variability can be used to estimate the accuracy of the assay, though any estimate will be inflated by intra-plate variation and this must be taken into account. The intra- and inter-plate variances in both assays were determined here across a range of DNA concentrations and mtDNA deletion/copy number levels.

### Intra-plate variability of the mitochondrial *MT*-*ND1*/*MT*-*ND4* deletion assay is dependent on DNA concentration and heteroplasmy level

**Figure 1:**
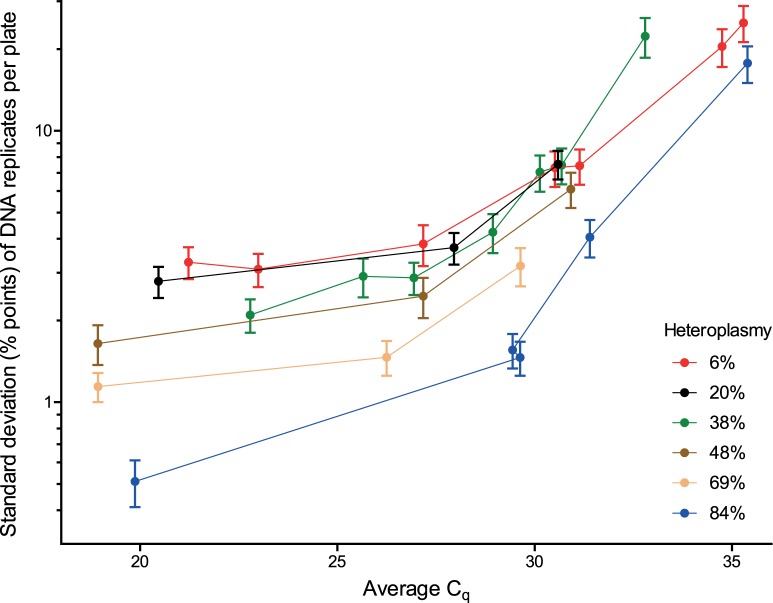
Effects of DNA concentration and heteroplasmy level on the accuracy of the *MT*-*ND1*/*MT*-*ND4* deletion assay. Percentage standard deviations of DNA sample replicates (*n* = 84) are represented relative to average C_q_ for homogenate skeletal muscle DNA samples (*n* = 6) varying in *MT*-*ND4* deletion levels from 10–80%. Confidence intervals at 95% are shown. The y axis is log_10_ scaled.

**Figure 2:**
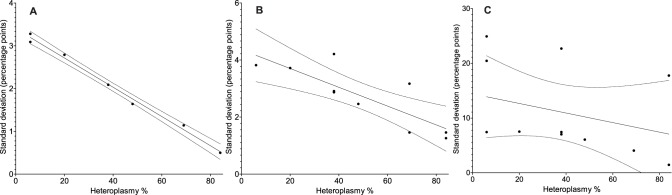
Relationship between *MT-ND1*/*MT-ND4* deletion assay variability and heteroplasmy level at high, moderate and low DNA concentrations. For all panels, percentage point standard deviations of DNA sample replicates (*n* = 84) are displayed relative to deletion heteroplasmy in homogenate skeletal muscle samples. Regression lines (solid) and 95% confidence intervals for the regression lines (dotted lines) are shown. **A** Linear relationship (*n* = 7, r = −0.997, P<0.0001) between the percentage MT-ND4 deletion level (heteroplasmy) and *MT-ND1*/*MT-ND4* deletion assay variation at high DNA concentrations (average C_q_ values for all DNA samples below 25). **B** A similar linear relationship (*n* = 10, r = −0.831, P = 0.0029) is seen at moderate DNA concentrations (average C_q_ values for all DNA samples between 25 and 30). **C** At low DNA concentrations (average C_q_ values for all DNA samples above 30) there is not a significant linear trend (*n* = 11, r = −0.209, P = 0.5368).

*MT-ND1*/*MT-ND4* deletion assay intra-plate variance was assessed using DNA samples from six patients (patients 1–6) harbouring single, large-scale mtDNA deletions with varying *MT-ND4* deletion levels (6–84%). This analysis identified two factors that were affecting the precision of the assay, namely DNA concentration and level of mtDNA deletion heteroplasmy (Figure 1). For each DNA sample analysed, the precision of the mtDNA deletion measurement decreased consistently with decreasing DNA concentration (Spearman’s r = 0.83, *n* = 28, P<0.0001) and assay variability became notably high at DNA concentrations associated with C_q_>30 (equivalent to a final PCR DNA concentration of less than 0.01 pg/µl). Furthermore, the precision of the *MT-ND1*/*MT-ND4* assay decreased with decreasing sample deletion levels. Specifically, at high DNA concentrations (C_q*MT-ND1*_<25; [DNA]_PCR_>0.3 pg/µl), a linear inverse relationship between the level of mtDNA heteroplasmy in a sample and assay variability was identified (*n* = 7, r = −0.993, P<0.0001; Figure 2). A similar linear relationship was observed at moderate DNA concentrations (25<C_q*MT-ND1*_<30; *n* = 10, r = −0.824, P = 0.0033). At low DNA concentrations (C_q*MT-ND1*_>30) no linear relationship was apparent (*n* = 11, r = −0.326, P = 0.3005).

### Inter-plate variability of the mitochondrial *MT-ND1*/*MT-ND4* deletion assay is low

**Figure 3:**
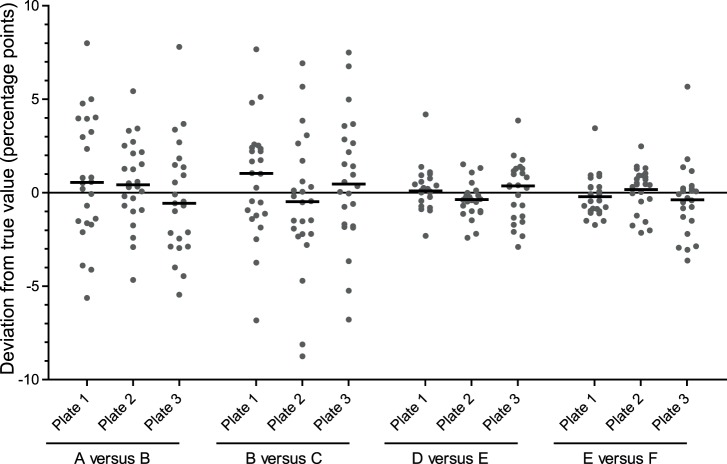
Inter- and intra-plate variability in the measurement of *MT*-*ND4* deletion level differences between DNA samples. Independent values for the difference in deletion level (in percentage points) between two homogenate skeletal muscle DNA samples harbouring either low (20–25%; samples A–C) or high (70–80%; samples D–F) *MT*-*ND4* deletion levels are shown for each sample replicate (*n* = 24; grey dots) per replicate real time PCR plate (*n* = 3). Data are normalised by subtracting the best estimate of the true deletion level difference, determined as the mean of all replicates on all plates. Mean values for each dataset are displayed (horizontal bars). The mean values from individual plates are within 1% of the actual deletion level, though individual replicates vary by up to ±9% at low deletion level and ±6% at high deletion level.

In view of the effect of heteroplasmy on intra-plate variability, inter-plate variance was assessed on two sets of DNA samples at high DNA concentration, one set harbouring low levels of deletion (20–25%, samples A–C) and the other set high levels (70–80%, samples D–F) (Figure 3). Independent values for the differences in deletion level between sets of two samples (A versus B and B versus C, D versus E and E versus F) were calculated for each replicate (*n* = 24) per plate (*n* = 3). The true deletion level difference was estimated for each pair of samples from the mean of all replicates across all plates. The deviation of these independent values from the best estimate of the true deletion level difference was then determined. The estimate for the inter-plate standard deviation at low deletion level was 0.496% and 0.4795% at high deletion level, with a pooled estimate of 0.485% using all samples. Interestingly, the inter-plate variation was not found to be substantially affected by the deletion level of the samples under comparison. Although Figure 3 suggests that the low deletion level samples exhibited higher inter-plate variation, this extra variability is almost entirely attributable to the higher intra-plate variation.

### DNA concentration also affects the intra-plate variability of the mtDNA copy number assay

**Figure 4:**
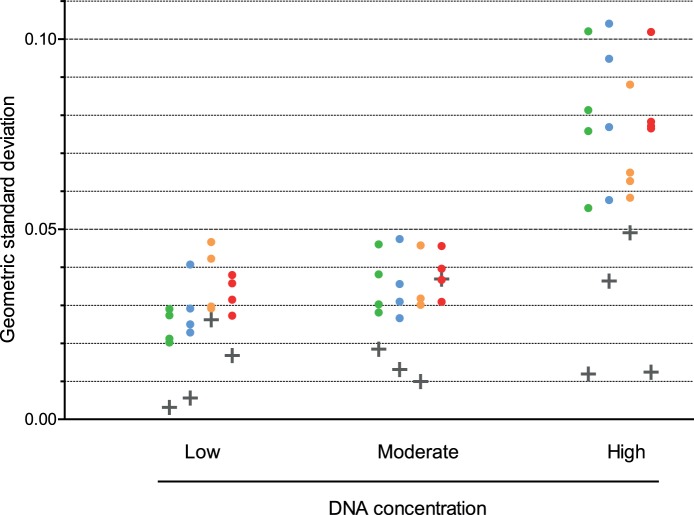
Inter- and intra-plate variability in the measurement of mtDNA copy number differences between DNA samples. Replicate (*n* = 12) *MT*-*ND1*/*B2M* copy number ratios were obtained for five homogenate skeletal muscle DNA samples of varying mtDNA copy number (samples S1 to S5) on each of four replicate *MT*-*ND1*/*B2M* real time PCR plate pairs at different DNA concentrations (low – corresponding to an average C_q*MT-ND1*_ of 19 and an average C_q*B2M*_ of 27, moderate - C_q*MT-ND1*_ = 24 and C_q*B2M*_ = 30, high - C_q*MT-ND1*_ = 25.5 and C_q*B2M*_ = 33). Independent relative copy number values were then calculated for each replicate of S2, S3, S4 and S5 compared to S1 for each *MT*-*ND1*/*B2M* plate. Intra-plate standard deviations are shown as circles (relative copy number values: S2:S1 = 1.10 (green circles), S3:S1 = 1.25 (blue circles), S4:S1 = 1.50 (orange circles), S5:S1 = 1.9 (red circles)). Inter-plate standard deviations are shown as grey crosses. As relative copy number is a multiplicative relationship, geometric standard deviations are shown.

Five DNA samples (S1–S5) of varying mtDNA levels were used to assess the variability of the *MT*-*ND1*/*B2M* assay. Replicate *MT*-*ND1*/*B2M* copy number ratios (*n* = 12) were generated for each sample from 4 replicate plate pairs at different DNA concentrations. Independent relative copy number values were then calculated for each plate pair replicate of S2, S3, S4 and S5 compared to S1. As relative copy number is a ratio, intra-plate and inter-plate variability were quantified as geometric standard deviations. Similar to the mtDNA deletion assay, intra-plate variability in the mtDNA copy number assay was dependent on C_q_, with variability increasing with increasing C_q_ (Spearman’s r = 0.81, *n* = 48, *P*<0.0001; Figure 4). Intra-plate variability was not however related to copy number ratio with dilute DNA samples (C_q*MT*-*ND1*_>25, [DNA]_PCR_<0.3 pg/µl; r = 0.031, *n* = 16, *P* = 0.9084), moderate DNA concentrations (C_q*MT*-*ND1*_ = 24, [DNA]_PCR_ = 0.6 pg/µl; r = 0.167, *n* = 16, *P* = 0.5373) or concentrated DNA samples (C_q*MT*-*ND1*_<20, [DNA]_PCR_>10 pg/µl; r = 0.423, *n* = 16, *P* = 0.1025). The estimates for the intra-plate geometric standard deviation are 0.0686 (corresponding scale factor 1.071; high DNA concentration), 0.0834 (1.087; moderate) and 0.181 (1.199; low).

Inter-plate variability was not significantly related to C_q_ (Spearman’s r = 0.26, *n* = 12, *P* = 0.2578), nor was there a significant linear relationship between inter-plate variability and mtDNA copy number ratio (r = 0.284, *n* = 12, P = 0.3685). The estimate of inter-plate geometric standard deviation calculated from all samples is 0.0305 (scale factor 1.031), which is lower than the intra-plate standard deviation at all DNA concentration levels.

### Recommendations on replicate numbers for the mtDNA deletion and copy number assays

Based on the data presented, it is possible to determine the numbers of replicates of samples and real-time PCR plates required to achieve specific levels of accuracy with the two mtDNA assays. Recommendations on the number of *MT-ND1*/*MT-ND4* sample and plate replicates necessary to confidently determine a given heteroplasmy difference (1–20%) between two homogenate skeletal muscle DNA samples are provided in Table 2. Table 3 outlines replicate numbers necessary to identify a specific difference (10–50%) in mtDNA copy number between two DNA samples at different DNA concentrations. The methodology to calculate sample sizes and all the required parameters for determining replicate numbers at other mtDNA deletion/copy number levels are provided in File S1.

**Table 2:** Number of plates required to identify a given heteroplasmy difference between two homogenate skeletal muscle DNA samples at various *MT*-*ND4* deletion levels with the *MT*-*ND1*/*MT*-*ND4* Taqman deletion assay.

			Detectable difference in mtDNA deletion level									
			1%		2%		5%		10%		20%	
Replicates per plate	Deletion Level	SD	P	*n*	P	*n*	P	*n*	P	*n*	P	*n*
3	10%	1.83%	0.80	54	0.82	15	0.89	4	>.99	3	>.99	2
3	20%	1.64%	0.81	44	0.81	12	0.95	4	0.84	2	>.999	2
3	30%	1.45%	0.81	35	0.83	10	0.88	3	0.91	2	>.999	2
3	40%	1.27%	0.81	27	0.83	8	0.94	3	0.95	2	>.999	2
3	50%	1.09%	0.81	20	0.82	6	0.98	3	0.98	2	>.999	2
3	60%	0.92%	0.82	15	0.85	5	>.99	3	>.99	2	>.999	2
3	70%	0.76%	0.84	11	0.87	4	0.89	2	>.999	2	>.999	2
3	80%	0.62%	0.85	8	0.84	3	0.96	2	>.999	2	>.999	2
3	90%	0.52%	0.85	6	0.93	3	0.99	2	>.999	2	>.999	2
6	10%	1.34%	0.81	30	0.84	9	0.92	3	0.94	2	>.999	2
6	20%	1.21%	0.80	24	0.81	7	0.96	3	0.97	2	>.999	2
6	30%	1.08%	0.81	20	0.82	6	0.98	3	0.99	2	>.999	2
6	40%	0.96%	0.81	16	0.82	5	>.99	3	>.99	2	>.999	2
6	50%	0.84%	0.83	13	0.91	5	0.83	2	>.999	2	>.999	2
6	60%	0.73%	0.82	10	0.89	4	0.90	2	>.999	2	>.999	2
6	70%	0.64%	0.83	8	0.82	3	0.95	2	>.999	2	>.999	2
6	80%	0.56%	0.80	6	0.90	3	0.98	2	>.999	2	>.999	2
6	90%	0.50%	0.87	6	0.95	3	0.99	2	>.999	2	>.999	2

Standard deviation (SD) is expressed in percentage points.

Actual power (P) and number of plates (*n*) are shown. C_q_<23 ([DNA]_PCR_>1 pg/µl).

**Table 3:** Number of *MT*-*ND1* and *B2M* real time PCR plates required to detect a specific relative difference in mtDNA copy number between two homogenate skeletal muscle DNA samples for a given number of replicate sample wells.

		Detectable difference in mtDNA copy number									
		10%		20%		30%		40%		50%	
Replicatesper plate	DNA Concentration	P	*n*	P	*n*	P	*n*	P	*n*	P	*n*
3	High	0.80	6	0.88	3	0.99	3	0.87	2	0.95	2
3	Moderate	0.84	8	0.94	4	0.97	3	1.00	3	0.90	2
3	Low	0.80	25	0.82	8	0.87	5	0.92	4	0.88	3
6	High	0.83	5	0.95	3	0.81	2	0.94	2	0.98	2
6	Moderate	0.85	6	0.91	3	1.00	3	0.90	2	0.96	2
6	Low	0.81	16	0.87	6	0.91	4	0.91	3	0.97	3

Each cell defines the number of plates (*n*) and actual power (P; minimum power 0.8 at α = 0.05) for a two sample *t*-test to detect, with the specified number of sample replicates per plate, a given percentage difference in mtDNA copy number. Estimates are calculated at high (approximate C_q*MT-ND1*_ of 19 ([DNA]_PCR_ = 20 pg/µl) and C_q*B2M*_ of 27 ([DNA]_PCR_ = 0.6 ng/µl)), moderate (approximate C_q*MT-ND1*_ of 24 ([DNA]_PCR_ = 0.6 pg/µl) and C_q*B2M*_ of 30 ([DNA]_PCR_ = 0.1 ng/µl)) and low (approximate C_q*MT-ND1*_ of 25 ([DNA]_PCR_ = 0.3 pg/µl) and C_q*B2M*_ of 33 ([DNA]_PCR_ = 10 pg/µl)) PCR DNA concentration levels.

## Discussion

The purpose of this study was to comprehensively assess the variability present within two quantitative real-time PCR assays, the *MT*-*ND1*/*MT*-*ND4* multiplex mtDNA deletion assay [11] and a *MT*-*ND1*/*B2M* singleplex mtDNA copy number assay [19], and provide guidelines for achieving accurate and reliable heteroplasmy and copy number measurements in homogenate skeletal muscle samples across a range of experimental conditions (DNA concentration and deletion level/relative copy number). The precision of both assays was found to be dependent on DNA concentration; mtDNA deletion level, but not relative copy number, also affected assay variability. Accurate mtDNA measurements are essential for detecting small differences in mtDNA quantity and integrity, critical for improving our understanding of mtDNA disease progression and evaluating the efficiency of novel therapeutic approaches. To the best of our knowledge, this is the first study to detail necessary sample and real-time PCR plate replicates to determine a given difference in mtDNA deletion levels and copy number.

There are many published methods for quantifying mtDNA, all targeting different mitochondrial and nuclear genomic regions and relying on different real-time chemistries [12], [14]–[17], [23], [24]. However, few researchers have reported how the variability of their assays is affected by the experimental conditions. Until now, only a cursory assessment of the reproducibility of our *MT*-*ND1*/*MT*-*ND4* deletion assay had been carried out [11], which, coupled to a growing lack of confidence in the assay’s ability to accurately measure deletion levels below 20–30% [9], [25], has been limiting the scope of the assay. The additional sample processing and pipetting associated with the singleplex approach of the mtDNA copy number assay also necessitate a comprehensive analysis of this assay’s intrinsic error. These particular assays were chosen for evaluation as we believe they offer versatility and flexibility with regards to mtDNA deletion level and copy number quantification. The deletion assay targets a highly conserved *MT*-*ND1* genomic region as well as an mtDNA region absent in over 95% of all reported deleted molecules (*MT*-*ND4*), a design which permits the detection of the vast majority of mtDNA deletions described to date [26]. Furthermore, the assay can be applied to both homogenate tissue and single cell investigations [7], [9], [11], [18], [27], [28]. The mtDNA copy number assay uses Taqman chemistry to ensure specificity and maintain compatibility with the deletion assay, enabling *MT*-*ND1* data to be shared between both quantification assays. The assay exploits a single-copy nuclear gene, *B2M*, which, unlike other often used reference genes (e.g. *ACTB* [29] or 18S rRNA [30]), has low sequence variability ([12]; Table S1) and permits inter-patient comparisons. Finally, the singleplex design not only circumvents the issue of reagent saturation associated with multiplexing, but also provides the added benefit of allowing the sample DNA concentration to be modified in order to maintain nuclear quantification cycles below 30.

The ability to optimise the sample DNA concentration is particularly important in view not only of the recent publication by Sochivko *et al.*, which describes the dependency of real-time PCR measurement error on DNA concentration [20], but also of our present data, which similarly demonstrate how decreasing sample concentration increases variability in both the *MT*-*ND1*/*MT*-ND4 and *MT*-*ND1*/*B2M* assays. With homogenate skeletal muscle samples, we highly recommend final DNA concentrations in *B2M* reactions be kept above 0.1 ng/µl (equivalent to C_q*B2M*_<30), whilst in the *MT*-*ND1*/*MT*-*ND4* reactions, final DNA concentrations between 0.1 ng/µl and 0.3 pg/µl (equivalent to 16.5<C_q*MT-ND1*_<25) are optimal. In our experience, 10 mg of biopsy tissue provide ample material to perform the necessary numbers of repeats required for the highest level of accuracy we believe to be achievable with both mtDNA assays.

Much interest is now placed on elucidating molecular events at a single cell level. Although beyond the scope of this study, the level of accuracy to be expected in single cell measurements can be extrapolated. Based on the assumption a single cell contains 6.5 pg genomic DNA, it should be possible to perform up to 10 repeat measurements of *MT*-*ND1* and *MT*-*ND4* with a final PCR DNA concentration approximating 0.03 pg/µl, achieving C_q_ values just below 30, the critical threshold above which assay variation increases exponentially (although the *MT*-*ND4* C_q_ value could be higher depending on the level of mtDNA deletion present and the mtDNA copy number will vary depending on cell type). Single cell analyses will inevitably be limited by a higher level of noise within the data and small differences in deletion level or copy number will not be accurately detected with current real-time PCR technology.

Consistent with the preliminary assessment of the multiplex *MT*-*ND1*/*MT*-*ND4* assay [11], we have confirmed a relationship between sample heteroplasmy and assay accuracy; as deleted mtDNA levels decrease, measurement error also increases. Despite this increased error, we have confirmed the *MT*-*ND1*/*MT*-*ND4* assay can be used to detect deletion levels below the previously published 20–30% cut-off threshold [9], [25], with an accuracy comparable to that achieved with high deletion load samples, providing sufficient replicates are included in the analysis.

Contrary to the observed effect of heteroplasmy on the deletion assay, variation in the mtDNA copy number assay was found to be independent of the relative difference in mtDNA abundance, within the range investigated (a maximum of approximately 2-fold difference). Furthermore, despite expectations that the extra DNA dilution for the *MT*-*ND1* measurement may have introduced an unacceptable level of experimental error into the procedure, the inter-plate variability (1.031 scale factor), which would incorporate this error, was found to be lower than the intra-plate variability at all DNA concentration levels. This is in contrast to reports from Malik and colleagues, who described a ‘dilution bias’ when measuring blood mtDNA copy number at different DNA concentrations [31]. Analysis of a different tissue, skeletal muscle, which harbours up to two orders of magnitude more mtDNA than blood, and the development of a rigorous protocol for diluting the DNA samples and performing the real-time PCR analysis may in part explain how dilution effects were minimised in this study.

In summary, we have devised guidelines regarding the number of sample and real-time PCR plate replicates necessary for accurately and reliably determining a given difference in mtDNA integrity and abundance in homogenate skeletal muscle samples at different DNA concentrations and mtDNA deletion/copy number levels. Researchers routinely only include three sample replicates in any quantitative PCR analysis and only perform up to three replicate plates; it is clear from this study however that this approach is not always valid, in particular at low level deletion and low DNA concentration with the *MT*-*ND1*/*MT*-*ND4* assay and with the mtDNA copy number assay in general. By defining the limits of detection of these two assays, it should now be possible to investigate, with greater accuracy, the molecular consequences of novel therapeutic approaches for mitochondrial disease. Moreover, we believe these recommendations will also help advance our current understanding of the clinical progression of mitochondrial pathologies.

## Supporting Information

Figure S1 **DNA integrity demonstrated by agarose gel electrophoresis.** In order to ensure storage at 4°C did not impact on the integrity of the DNA, available DNA samples (50–100 ng) were analysed by electrophoresis through a 1% agarose gel before (1) and after (2) storage for up to a month at 4°C. L, Promega 1 kb DNA ladder.
(DOC)

Table S1 **Sequence variability of commonly used real-time PCR reference genes.** Data obtained from NCBI dbSNP database (Sherry et al. (2001) Nucleic Acids Res. 29(1):308-11) in September 2014.
(DOC)

File S1 **Methodology for calculating replicate sample and plate numbers.**
(DOC)
