# Supplementary material for: Accurate Measurement of Mitochondrial DNA Deletion Level and Copy Number Differences in Human Skeletal Muscle
Source: PLoS One. 2014 Dec 4;9(12):e114462. doi: 10.1371/journal.pone.0114462 (PMC4256439; doi:10.1371/journal.pone.0114462)
Supplement: Figure S1 — DNA integrity demonstrated by agarose gel electrophoresis. In order to ensure storage at 4°C did not impact on the integrity of the DNA, available DNA samples (50–100 ng) were analysed by electrophoresis through a 1% agarose gel before (1) and after (2) storage for up to a month at 4°C. L, Promega 1 kb DNA ladder. (DOC) [file pone.0114462.s001.doc]

**Figure S1. DNA integrity demonstrated by agarose gel electrophoresis.**

In order to ensure storage at 4°C did not impact on the integrity of the DNA, available DNA samples (50-100ng) were analysed by electrophoresis through a 1% agarose gel before (1) and after (2) storage for up to a month at 4°C. L, Promega 1kb DNA ladder.

**
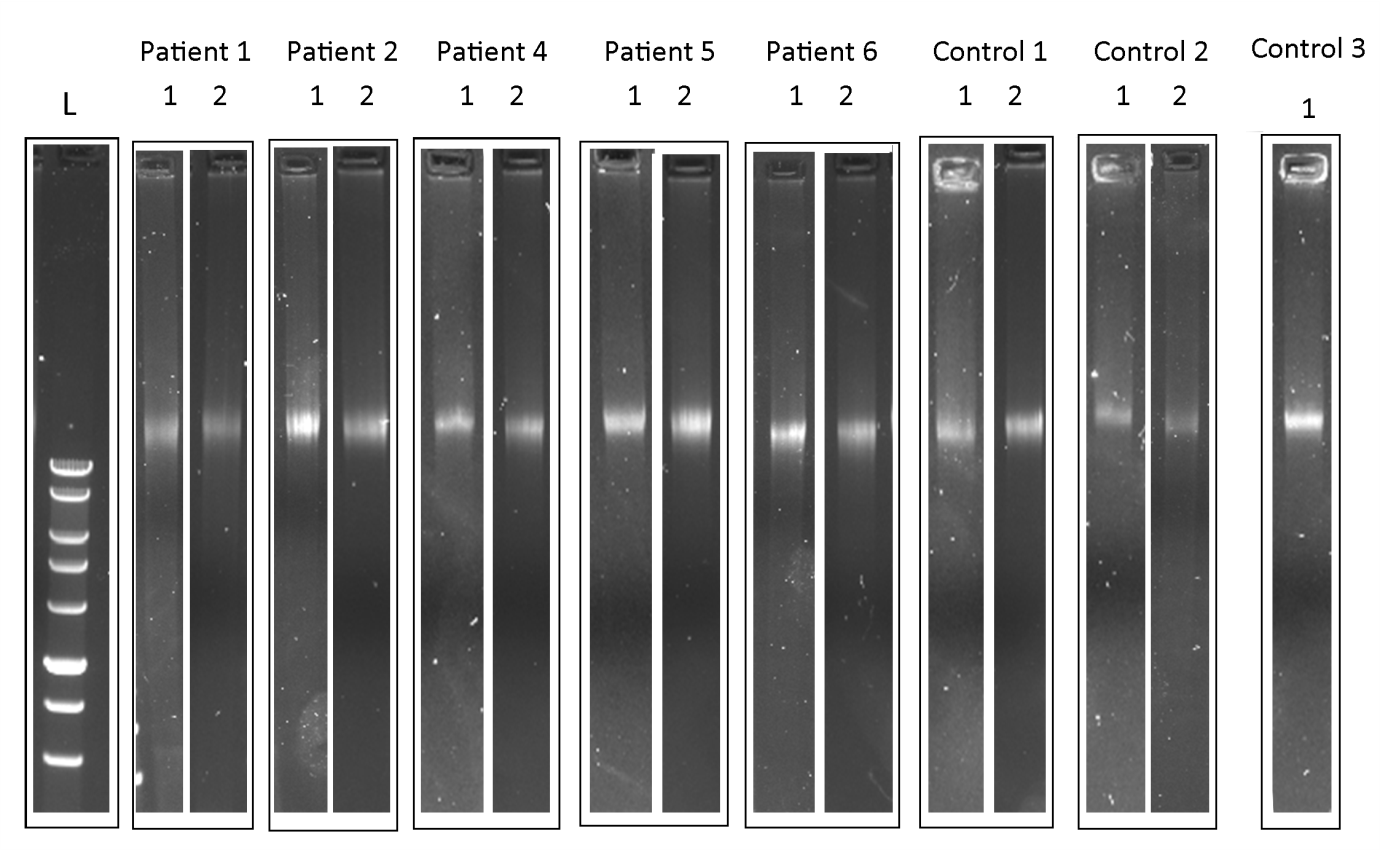
**
