# Supplementary material for: Accurate Measurement of Mitochondrial DNA Deletion Level and Copy Number Differences in Human Skeletal Muscle
Source: PLoS One. 2014 Dec 4;9(12):e114462. doi: 10.1371/journal.pone.0114462 (PMC4256439; doi:10.1371/journal.pone.0114462)
Supplement: Table S1 — Sequence variability of commonly used real-time PCR reference genes. Data obtained from NCBI dbSNP database (Sherry et al. (2001) Nucleic Acids Res. 29(1):308-11) in September 2014. (DOC) [file pone.0114462.s002.doc]

**Table S1. Sequence variability of commonly used real-time PCR reference genes.** Data obtained from NCBI dbSNP database (Sherry *et al.* (2001) Nucleic Acids Res. 29(1):308-11) in September 2014.

| Gene | Number of coding SNP loci | Coding region size (bp) | Number of SNP loci/100 bases |
| --- | --- | --- | --- |
| *GAPDH* | 94 | 1513 | 6.2 |
| *ACTB* | 81 | 1852 | 4.37 |
| *B2M* | 18 | 987 | **1.8** |
| *RPPH1* | 23 | 341 | 6.7 |
| *LPL* | 78 | 3747 | 2.1 |
| *RNA18S5* | N/A |  |  |
