# Supplementary material for: Accurate Measurement of Mitochondrial DNA Deletion Level and Copy Number Differences in Human Skeletal Muscle
Source: PLoS One. 2014 Dec 4;9(12):e114462. doi: 10.1371/journal.pone.0114462 (PMC4256439; doi:10.1371/journal.pone.0114462)
Supplement: File S1 — Methodology for calculating replicate sample and plate numbers. (DOC) [file pone.0114462.s003.doc]

**File S1 – Methodology for calculating replicate sample and plate numbers.**

**General Guidance**

Tables 2 and 3 in the manuscript provide sample sizes required to identify a common set of deletion level and copy number changes that are anticipated to be useful. However, the information in Supporting Information 1 and 2 can be used to calculate the number of repeat plates required to detect a chosen deletion level difference or relative copy number for any deletion level or copy number to any required precision.

Both processes involve consideration of the intra-plate variation nested experimentally within the inter-plate variation. The first step is to choose the number of replicates of each sample to run per plate; where intra-plate variation is high it can be more efficient to increase the replicates per plate rather than increasing the number of plates. This must be ascertained empirically using the data and procedure documented below, though Tables 2 and 3 in the manuscript will provide broad guidance on how many replicates per plate would be most efficient.

Given a number of replicates per plate, the resultant experimental standard deviation can be calculated using the following formula:


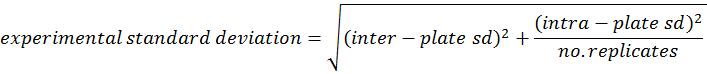


If running multiple samples at different deletion levels, it is feasible to use a higher number of replicates per plates for low deletion level samples.

**1.1 Parameter estimates for the linear relationship between the standard deviation and *MT-ND4* deletion levels at high (C_q_ < 25, [DNA]_PCR_ > 0.3pg/µl) and moderate (25 < C_q_ < 30, 0.3pg/µl > [DNA]_PCR_ > 0.01pg/µl) PCR DNA concentration levels, and methodology to calculate sample sizes.**

| DNA concentration | Parameter | Estimate | 95% Lower | 95% Upper |
| --- | --- | --- | --- | --- |
| High | Slope | -0.03421 | -0.03761 | -0.03018 |
| High | Intercept | 3.400% | 3.237% | 3.563% |
| Moderate | Slope | -0.03294 | -0.05138 | -0.01450 |
| Moderate | Intercept | 4.361% | 3.340% | 5.383% |
| Low | Median | 7.445% | 6.3% | 19.8% |

For low DNA concentrations, the linear regression is not significant, and thus the estimated median standard deviation is shown with 95% confidence intervals.

Inter-plate standard deviation is 0.485% for all deletion levels and DNA concentrations.

To calculate the number of plates required to detect a given deletion level difference:

1. For a given deletion level and DNA concentration, use the regression parameters to calculate the intra-plate standard deviation. e.g. for 60% deletion level at high DNA concentration, the standard deviation is
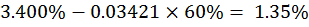
.
2. Calculate the overall standard deviation resulting from the nested intra-plate variation within the inter-plate variation, e.g. for 3 and 6 replicates per plate with the same example of 60% deletion level at high DNA concentration the resulting standard deviations are


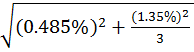
=0.918% and
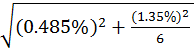
=0.734% respectively.

1. Use a sample size calculator (found in many stats applications, or online calculators are available) to calculate the sample size (number of repeat plates) required for a 2 sample t-test to identify the required difference in deletion level. E.g. to detect a 1% change in deletion level with the given experimental variation (standard deviation of 0.918% for 3 replicates per plate) at a chosen power level (usually 0.8), 15 plates are required at 3 replicates per plate, or 10 plates at 6 replicates per plate.

**1.2** Geometric standard deviations for the copy number assay and methodology to calculate sample sizes.

| DNA concentration | Intra-plate standard deviation | Intra-plate scale factor | Inter-plate  standard  deviation | Inter-plate scale  factor |
| --- | --- | --- | --- | --- |
| High | 0.0686 | 1.071 | 0.0305 | 1.031 |
| Moderate | 0.0834 | 1.087 | 0.0305 | 1.031 |
| Low | 0.181 | 1.199 | 0.0305 | 1.031 |

To calculate the number of plates required to detect a given copy number change:

1. Calculate the overall standard deviation resulting from the nested intra-plate variation within the inter-plate variation, e.g. for 3 and 6 replicates per plate at high DNA concentration, the resulting standard deviations are


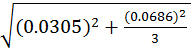
=0.0500 and
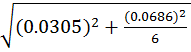
=0.0414 respectively.

1. Calculate the log_e_ of the required percentage change you wish to be able to measure. For example, a 10% change would be log_e_(1.10) = 0.09531.
2. Use a sample calculator (found in many stats applications, or online calculators are available) to calculate the sample size (number of repeat plates) required for a 2 sample t-test to identify the given difference (in this case 0.09531) with the given standard deviation (e.g. 0.0500 for 3 replicates per plate) at a chosen power level (usually 0.8). In this case the result is 6 plates.
